# Matrix Analysis of Warped Stretch Imaging

**DOI:** 10.1038/s41598-017-11238-5

**Published:** 2017-09-11

**Authors:** Chanju Kim, Ata Mahjoubfar, Jacky C. K. Chan, Akio Yazaki, Young-Chul Noh, Bahram Jalali

**Affiliations:** 1 Advanced Photonics Research Institute, GIST, Gwangju, 61005 Republic of Korea; 2 Department of Electrical Engineering, University of California, Los Angeles, California 90095 USA; 3 California NanoSystems Institute, Los Angeles, California 90095 USA; 4 Yokohama Research Laboratory, Hitachi, Ltd., Kanagawa, 244-0817 Japan; 5 Department of Bioengineering, University of California, Los Angeles, California 90095 USA

## Abstract

Sensitive and fast optical imaging is needed for scientific instruments, machine vision, and biomedical diagnostics. Many of the fundamental challenges are addressed with time stretch imaging, which has been used for ultrafast continuous imaging for a diverse range of applications, such as biomarker-free cell classification, the monitoring of laser ablation, and the inspection of flat panel displays. With frame rates exceeding a million scans per second, the firehose of data generated by the time stretch camera requires optical data compression. Warped stretch imaging technology utilizes nonuniform spectrotemporal optical operations to compress the image in a single-shot real-time fashion. Here, we present a matrix analysis method for the evaluation of these systems and quantify important design parameters and the spatial resolution. The key principles of the system include (1) time/warped stretch transformation and (2) the spatial dispersion of ultrashort optical pulse, which are traced with simple computation of ray-pulse matrix. Furthermore, a mathematical model is constructed for the simulation of imaging operations while considering the optical and electrical response of the system. The proposed analysis method was applied to an example time stretch imaging system via simulation and validated with experimental data.

## Introduction

Time stretch imaging technology enables fast continuous blur-free acquisition of quantitative phase and intensity image, offering high throughput image analysis for various applications such as industrial and biomedical imaging^1–11^. The imaging technology exploits spatial and temporal dispersion of broadband optical pulses, which realizes frame rates equivalent to the pulse repetition rates of mode-locked laser ranging from a few MHz to GHz^12^. The operating principle of the time stretch imager consists of the optical dispersion process in both space and time, the acquisition and the recording of optical scan signal, and reconstruction of image. First, spatial information of imaging target is mapped into spectrum of an ultrashort optical pulse (as a broadband flash illumination), which is realized with spatial dispersion. The optical spectrum is then mapped into a temporal waveform by temporal dispersion through a process known as time stretch transform^2, 3, 13–15^. The temporal data stream of encoded spatial information is serially collected and recorded by a single detection system consisting of a photodetector and a digitizer. Finally, the image is reconstructed by inversely mapping the recorded temporal data stream into the corresponding spatial coordinates.

A new advancement and variant of the time stretch imaging technology, warped stretch imaging^16, 17^ (also known as foveated time stretch imaging) features real-time optical compression of image while keeping the rapid imaging speed of its predecessor. Warped stretch imaging is realized by intentional warping of the formerly “linear” frequency-to-time mapping process with highly nonlinear temporal dispersion. The temporal stream of optical signal becomes a warped representation of the real image. Some portions of the temporal image signal are dilated in the time domain and other part are contracted. Optical image compression is effectively delivered by engineering the warp of the group delay dispersion profile, where higher image sampling densities are assigned to information-rich regions. Inspired by the sharp central vision (foveal vision) found in the human eye, where the photoreceptors (pixels) are concentrated in the center of gaze^18^ – warped stretch imaging is successfully demonstrated by real-time optical image compression in a time stretch infrared imaging system^16^ and an adaptive codec for digital image compression^19^.

In both conventional time stretch and warped stretch imaging systems, the optical front-end -- consisting of spatial and temporal dispersion elements -- is the significant and reconfigurable factor in designing the imaging operation. The optical dispersion encoding process in the front-end should be rigorously traced in space and time with respect to frequency (wavelength) for robust image construction and precise performance analysis of the imaging system. The time stretch process (frequency-to-time mapping) is typically performed within a single dispersive element. The process is theoretically well-established and experimentally reliable with the uniform form of time-stretch dispersive Fourier transform (TS-DFT) and nonuniform form of warped stretch transform^2, 13–15, 17, 20, 21^. In contrast, the one-dimensional spatial dispersion process (frequency-to-space mapping) is a relatively complicated step as it is achieved with combination of free-space optical components (i.e., diffraction grating and focusing lens), which there are a multitude of possible configurations. In addition, the nonlinear spatial dispersion present in both time stretch and warped stretch imaging systems should be considered as it has non-negligible warping effect in the mapping process. Due to such complexities in the frequency-to-space mapping process, it is a non-trivial task to establish a general description of the warped stretch imaging operation. We note that a theoretical analysis of time stretch imaging system has been formalized previously^22^. However, the study relies on an approximation of a system to a linear dispersion model which limits the accuracy of performance analysis and its use in image reconstruction. The only existing way to precisely analyze a warped stretch imaging system is by experimental observation of a constructed system^10, 11^.

To address these limitations, we introduce a numerical method aimed for analyzing arbitrary configurations of warped stretch imaging systems. The method allows accurate evaluation of frequency-space-time mappings and the simulation of complete imaging processes. Our analysis method is divided into two parts, consisting of (i) a modified matrix method and (ii) mathematical models of the components. The modified matrix method is built upon a ray-pulse matrix formalism developed by Kostenbauder^23^, which we modified to consider the nonlinear dispersion of the optical system. This allows for compact and comprehensive means of tracking the ray-pulse structure (frequency-space-time map) in an arbitrary free-space optical system by matrix computation. The mathematical model is constructed in order to numerically simulate the imaging process including the wave nature of the optical signal. We derive the mathematical expressions for each optical and electrical component in the system and incorporate the frequency-space-time map obtained from the modified matrix method. Employing our analysis method, we demonstrate numerical evaluation of important parameters such as field-of-view, number of pixels, reconstruction map, detection sensitivity and spatial resolution. In addition, we highlight the influence of nonlinear spatial dispersion on the acquired image. This demonstrates that the construction of warped stretch imaging can be extended to engineering spatial dispersion profiles. As seen in the studies of other imaging techniques^24, 25^, physically rigorous analysis should assist further development of the imaging system.

## Methods

### Principles of time/warped stretch imaging

**Figure 1:**
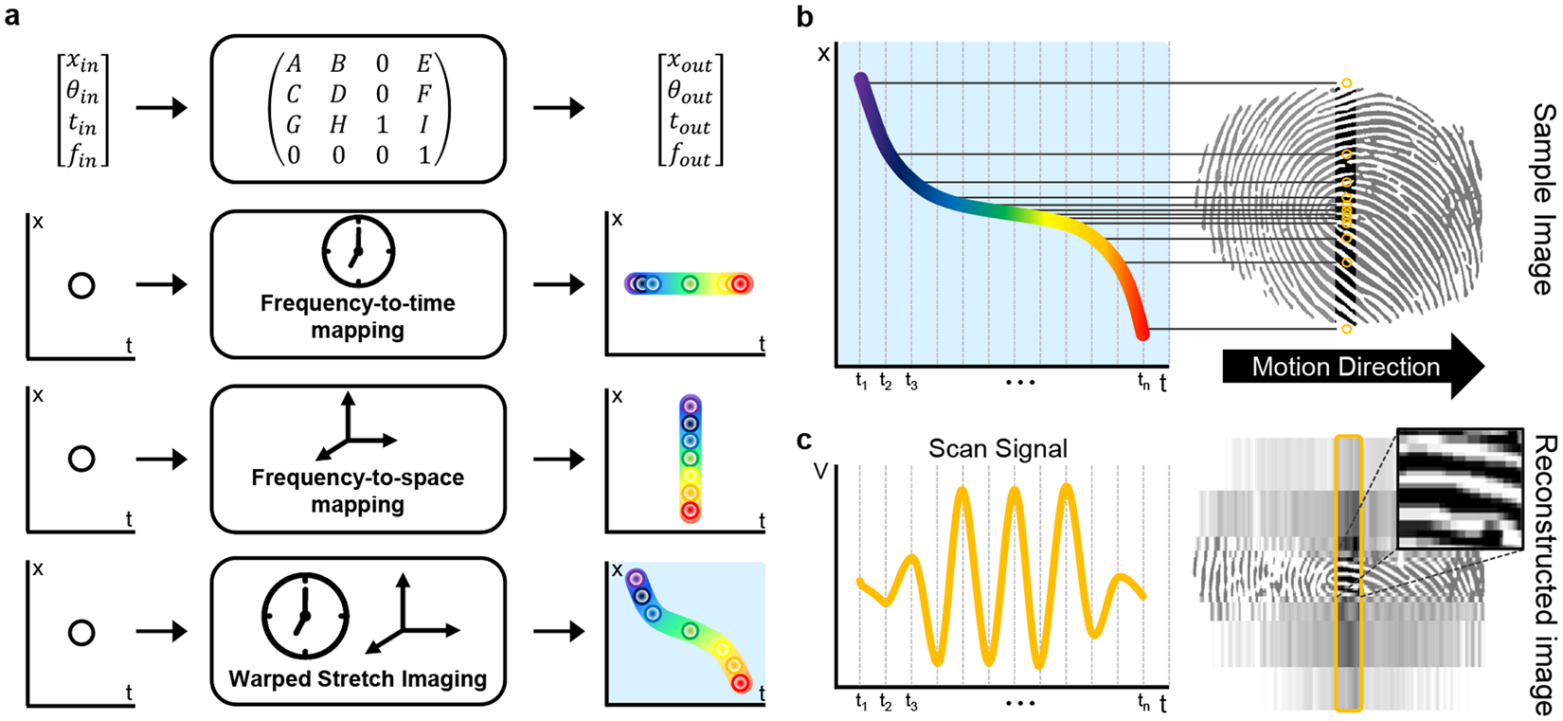
Principles of time/warped stretch imaging. **(a)** Time/warped stretch imaging employs frequency-to-time and frequency-to-space mapping of an ultrashort optical pulse to perform a line scan. Here, to clarify the method, we use a one-dimensional spatial disperser, which leads to a line scan per pulse. A 4 × 4 ray-pulse matrix computation of vector (space (*x*), slope (*θ*), time (*t*) and frequency (*f*)) specifies the optical mapping relationship. Colored circles represent uniformly spaced frequency components (beam-pulselets) and rainbow gradient lines represent their continuous distribution in both space and time domains. (**b**) Uniform temporal sampling under this space-to-time relationship determines the spatial sampling position on the image space. Yellow dots on the fingerprint sample image account for the warped pixel distribution of a given imaging system. (**c**) The ﻿corresponding temporal scan signal from the sampled points is acquired by a single-pixel photodetector and an analog-to-digital converter (ADC). The three pronounced peaks in the scan signal correspond to the fingerprint ridges in the center of the line scan. The scan signal is reconstructed by remapping the temporal data-stream back to the original image space. Regions with higher temporal dispersion are effectively assigned more samples (central region), while the part of the waveform that is not highly time-stretched corresponds to fewer imaging pixels (peripheral regions).

The key principle of time/warped stretch imaging is conceptually illustrated in Fig. 1a. Optical mapping processes distribute the frequency components of a broadband ultrashort optical pulse in space and time according to the optical components involved. (1) Frequency-to-time mapping projects the spectral information to the time domain and (2) frequency-to-space mapping encodes the spatial information into the spectrum. These two central optical processes can be cascaded regardless of their order, and enable the ultrafast acquisition of image in single-pixel scanner-like procedure. Figure 1a shows the arrangement of beam-pulselets for each mapping process in time and space, and the final space-time relationship of warped stretch imaging. The beam-pulselets can be addressed with their respective space (*x*), slope (*θ*), time (*t*) and frequency (*f*). The optical matrix computation yields the resultant inter-domain (space, time and frequency) relationship after the propagation. Here, the mapping relationships can be engineered by using optical dispersive elements of choice. We note that various optical elements were proposed and implemented since the advent of the time stretch imaging concept. Dispersive fibres, chirped fibre Bragg gratings, chromo-modal dispersion devices^26^ and free-space angular-chirped-enhanced delay devices^27^ can be used to define the frequency-to-time relationship, while diffraction gratings, diffractive optical elements, virtually imaged phased arrays, tilted fibre gratings and optical prisms are a few of available options for establishing the desired frequency-to-space relationship. As shown in Fig. 1b, the space-time relationship curve of warped stretched imaging system is uniformly point-sampled along the time axis, according to the fixed sampling rate of the ADC. Yellow colored circles on the image sample represent the spatially sampled points (pixels) corresponding to the discrete time domain of acquisition electronics. The warped optical mapping and pixel distribution of the acquired scan signal is highly foveated (Fig. 1c). The features in the center of the gaze are fully resolved, and the less important surrounding areas are imaged with lower resolution, reducing the required ADC sampling rate. Finally, the warped scan signal is reconstructed by remapping the temporal data-stream to the original image space according to the space-time relationship. We note that there are many variants of time/warped stretch imaging systems with different means of encoding spatial information and acquiring optical signals; however, they all follow the same mapping processes.

### Analysis Method

The imaging performance of time/warped stretch imaging systems is determined by the frequency-space-time mapping process, the electrical bandwidth and the overall sampling rate of the acquisition system, including photodetection and digitization^22^. In order to deliver a complete and accurate analysis of an imaging system, we devise an analysis method that replicates the imaging process by means of numerical simulation. Our analysis method consists of two sub-formalisms: (i) a modified matrix method and (ii) mathematical modeling. We are able to characterize the frequency-space-time relationship in an arbitrary time/warped stretch imaging system using the modified matrix method. Subsequently, the mathematical model incorporates the obtained mapping relationship with mathematical expressions for optical and electrical components. Numerical simulation of the mathematical model provides the basis of our analysis method.

In the following section, we first introduce the modified matrix method and validate the accuracy of the method by its application to a well-known laser pulse compressor. Second, we apply the matrix analysis to a time stretch imaging system where we obtain frequency-space-time mapping and important parameters such as field-of-view, number of pixels and image warp of the imager. Finally, we derive the mathematical model of time stretch imaging operation.

### Modified Matrix Method

The modified matrix method is based on a 4 × 4 optical matrix formalism developed by Kostenbauder which is widely used for characterizing spatiotemporally dispersive optical systems^23, 28^. The Kostenbauder matrix method uses vector with four elements [*x θ t f*]^*T*^ to define deviation of space, angle, time and frequency from the reference optical pulse. Here, the space and angle is designated to a transverse direction, and the time is determined in the propagating direction of the pulse. As shown in eq. (1), computing the product of an input vector *ν*_*in*_ = [*x*_*in*_ *θ*_*in*_ *t*_*in*_ *f*_*in*_]^*T*^ and a 4 × 4 optical matrix yields an output vector *ν*_*out*_ = [*x*_*out*_ *θ*_*out*_ *t*_*out*_ *f*_*out*_]^*T*^ which reflects the structure of the pulse after propagation.

1 $$[\begin{array}{c}{x}_{out}\\ {\theta }_{out}\\ {t}_{out}\\ {f}_{out}\end{array}]=(\begin{array}{cccc}A & B & 0 & E\\ C & D & 0 & F\\ G & H & 1 & I\\ 0 & 0 & 0 & 1\end{array})\cdot [\begin{array}{c}{x}_{in}\\ {\theta }_{in}\\ {t}_{in}\\ {f}_{in}\end{array}]$$

The 4 × 4 optical matrix in eq. (1) is able to model time invariant optical element or system with the convention:

2 $$(\begin{array}{cccc}A & B & 0 & E\\ C & D & 0 & F\\ G & H & 1 & I\\ 0 & 0 & 0 & 1\end{array})=(\begin{array}{cccc}\frac{\partial {x}_{out}}{\partial {x}_{in}} & \frac{\partial {x}_{out}}{\partial {\theta }_{in}} & 0 & \frac{\partial {x}_{out}}{\partial {f}_{in}}\\ \frac{\partial {\theta }_{out}}{\partial {x}_{in}} & \frac{\partial {\theta }_{out}}{\partial {\theta }_{in}} & 0 & \frac{\partial {\theta }_{out}}{\partial {f}_{in}}\\ \frac{\partial {t}_{out}}{\partial {x}_{in}} & \frac{\partial {t}_{out}}{\partial {\theta }_{in}} & 1 & \frac{\partial {t}_{out}}{\partial {f}_{in}}\\ 0 & 0 & 0 & 1\end{array})$$

where the constants A, B, C and D are identical to the elements of the ABCD ray matrix, E, F, G, H, and I represent the spatial dispersion, angular dispersion, pulse-front tilt, time-angle coupling, and group delay dispersion respectively. The optical matrices for conventional optical components such as free-space, lens, mirror, dispersive slab, prism and diffraction gratings are derived with simple geometric calculations^23^. We find that as the elements are constant values corresponding to first-order derivatives in the neighborhood of the given reference point, the optical matrix is accurate in dispersion up to the second order (linear dispersion). However, when imaging with broadband ultrashort pulses, higher-order dispersion should be considered in order to fully characterize an actual optical system. Therefore, we have constructed a generalized non-constant matrix as a modified form of the original Kostenbauder matrix. We distinguish our notation from the linear analysis where the modified vector is expressed as [Δ*x* Δ*θ* Δ*t* Δ*f*]^*T*^. With the following optical matrix, high-order spatial, angular and temporal dispersion (E, F, I) can be considered. We substitute the elements in the 4 × 4 optical matrix with frequency dependent functions as

3 $$M=(\begin{array}{cccc}A & B & 0 & \frac{{\rm{\Delta }}{x}_{out}({\rm{\Delta }}{f}_{in})}{{\rm{\Delta }}{f}_{in}}\\ C & D & 0 & \frac{{\rm{\Delta }}{\theta }_{out}({\rm{\Delta }}{f}_{in})}{{\rm{\Delta }}{f}_{in}}\\ G & H & 1 & \frac{{\rm{\Delta }}{t}_{out}({\rm{\Delta }}{f}_{in})}{{\rm{\Delta }}{f}_{in}}\\ 0 & 0 & 0 & 1\end{array})$$

The elements A, B, C, D, G and H are kept unchanged from the original form of Kostenbauder matrix (first-order derivative). In order to include high-order dispersion, E, F and I are now replaced as arbitrary function of Δ*f*_*in*_. The modified elements E, F and I, can be defined by inserting corresponding frequency-dependent function of an optical component. We provide matrices for the dispersive optical components in Table 1.

**Table 1:** Optical Matrices for diffraction grating and dispersive fiber.

Optical Component	Optical Matrix
Diffraction grating (*M*_*grating*_)	$$(\begin{array}{cccc}-\frac{\cos ({\rm{\Delta }}{\theta }_{out})}{\cos ({\rm{\Delta }}{\theta }_{in})} & 0 & 0 & 0\\ 0 & -\frac{\cos ({\rm{\Delta }}{\theta }_{in})}{\cos ({\rm{\Delta }}{\theta }_{out})} & 0 & -\frac{{\rm{\Delta }}{\theta }_{out}({\rm{\Delta }}{f}_{in})}{{\rm{\Delta }}{f}_{in}}\\ \frac{{\rm{\Delta }}{t}_{out}}{{\rm{\Delta }}{x}_{in}} & 0 & 1 & 0\\ 0 & 0 & 0 & 1\end{array})$$
	$$\frac{{\rm{\Delta }}{\theta }_{out}({\rm{\Delta }}{f}_{in})}{{\rm{\Delta }}{f}_{in}}=\frac{{\theta }_{d}({\rm{\Delta }}{f}_{in})-\theta (0)}{{\rm{\Delta }}{f}_{in}}$$
	$${\theta }_{d}({\rm{\Delta }}f)={\sin }^{-1}(\frac{c}{({f}_{0}+{\rm{\Delta }}f)d}-\,\sin \,{\theta }_{i})$$
	*θ*_*i*_: incident angle
	*θ*_*d*_: first-order diffraction angle
	*d*: grating density
	*c*: speed of light in vacuum
	*f*_0_: center frequency
Dispersive fiber (*M*_*fiber*_)	$$(\begin{array}{cccc}1 & 0 & 0 & 0\\ 0 & 1 & 0 & 0\\ 0 & 0 & 1 & \frac{{\rm{\Delta }}{t}_{out}({\rm{\Delta }}{f}_{in})}{{\rm{\Delta }}{f}_{in}}\\ 0 & 0 & 0 & 1\end{array})$$
	$$\frac{{\rm{\Delta }}{t}_{out}({\rm{\Delta }}{f}_{in})}{{\rm{\Delta }}{f}_{in}}=\frac{{\tau }_{g}({\rm{\Delta }}{f}_{in})-{\tau }_{g}(0)}{{\rm{\Delta }}{f}_{in}}$$
	*τ*_*g*_(Δ*f*): Group delay of dispersive fiber

An arbitrary optical system can be analyzed with the optical matrix calculation in a numerical fashion. The frequency components of the pulse are segmented over the whole spectrum as series of vectors where they are computed with the optical matrices. As a demonstration of our method, we analyzed a laser pulse compressor, a well-known and simple optical system consisting of diffraction grating pairs. Optical matrices for the laser pulse compressor are expressed as

4 $$[\begin{array}{c}{\rm{\Delta }}{x}_{out}\\ {\rm{\Delta }}{\theta }_{out}\\ {\rm{\Delta }}{t}_{out}\\ {\rm{\Delta }}{f}_{out}\end{array}]={M}_{grating}\cdot {M}_{freespace}\cdot {M}_{grating}\cdot {M}_{grating}\cdot {M}_{freespace}\cdot {M}_{grating}\cdot [\begin{array}{c}{\rm{\Delta }}{x}_{in}\\ {\rm{\Delta }}{\theta }_{in}\\ {\rm{\Delta }}{t}_{in}\\ {\rm{\Delta }}{f}_{in}\end{array}]$$

where ${\rm{\Delta }}{x}_{out}$ is the computed output vector with information of position, ${\rm{\Delta }}{\theta }_{out}$ is slope, ${\rm{\Delta }}{t}_{out}$ is time and ${\rm{\Delta }}{f}_{out}$ is frequency. Thus, the output vector completely defines the frequency-space-time relationship of the pulse after propagation through the system. Here, we have omitted the optical matrix for the free-space between the diffraction grating pairs, as it does not affect the temporal dispersion of a perfectly aligned pulse compressor.

**Figure 2:**
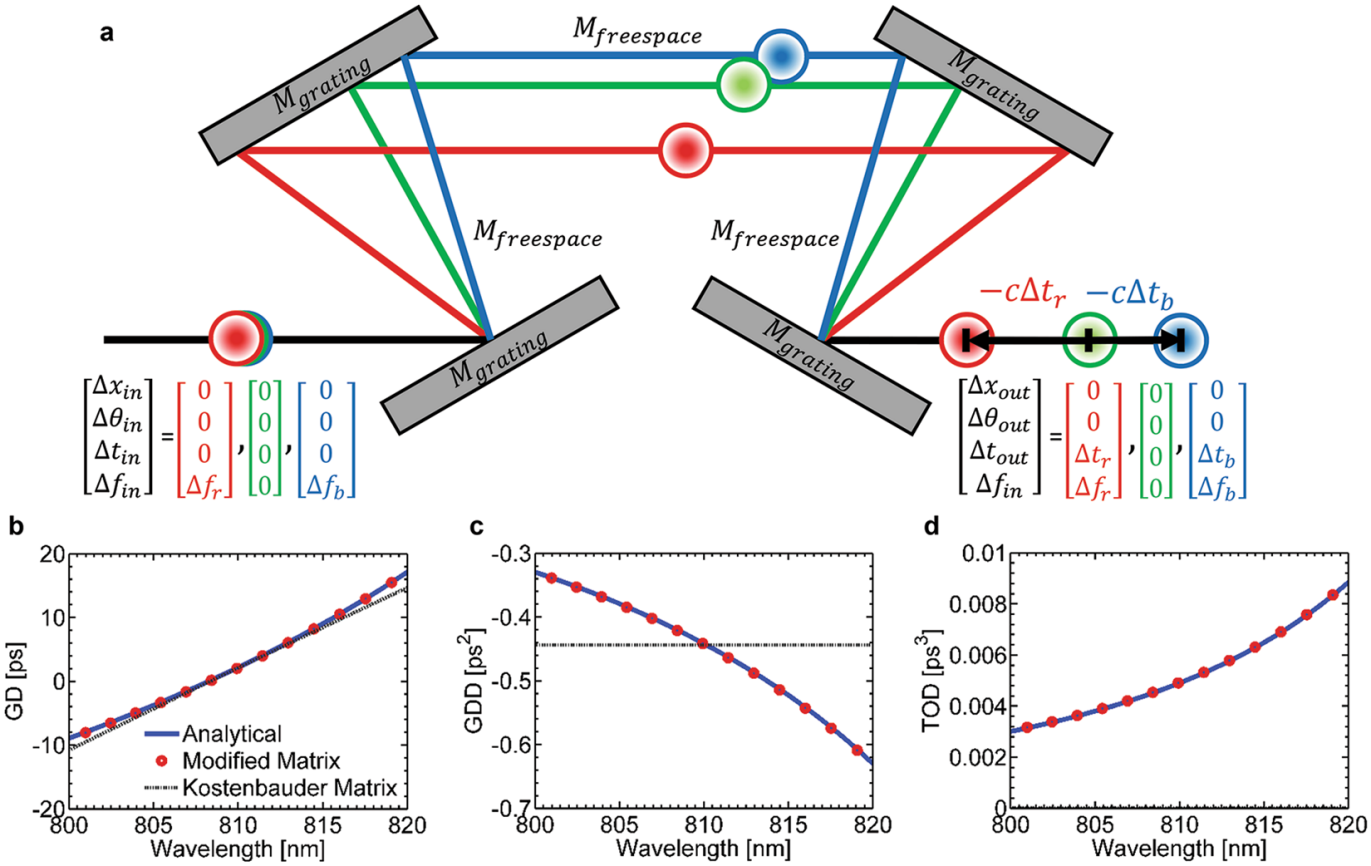
Demonstration of modified matrix analysis for laser pulse compressor. Red, blue and green line represent optical paths for each frequency component. **(a)** Schematic of laser pulse compressor. The center wavelength of the pulse is 810 nm, and the incident angle of the pulse at the first diffraction grating is 63 degrees (Littrow angle), and the separation distance between the diffraction grating pair is 1 cm. **(b)** Group delay, **(c)** group-delay dispersion, and **(d)** third-order dispersion of the laser pulse compressor evaluated by analytical formula (blue solid line), modified matrix (red circle) and original Kostenbauder matrix (grey dashed line).

To illustrate the method, the frequency components of the input pulse are segmented into three vectors as shown in Fig. 2a. The red, green and blue colors represent lowest frequency, reference (center) frequency and highest frequency respectively. We obtain three output vectors by computing the optical matrices with the three input vectors. As we analyze the output vectors of an ideal laser compressor, the deviation in space and slope in the output vectors are zero while the temporal deviation (group delay) are ${\rm{\Delta }}{t}_{r}$ 0, ${\rm{\Delta }}{t}_{b}$ for each frequency components (red, green and blue respectively). Using this convention, we acquire group delay profiles with sufficient number of frequency samples over the 20 nm optical bandwidth. We calculated the temporal dispersion and compared with the analytical result^29^ to validate our analysis method. The temporal dispersion of the system, group delay (GD), group delay dispersion (GDD) and third-order dispersion (TOD) can be evaluated by

5 $${\tau }_{g}(\omega )={\rm{\Delta }}{t}_{out}-\frac{d}{d\omega }[2\pi \frac{b}{d}\,\tan ({\theta }_{d})]$$

6 $$GDD(\omega )=\frac{d}{d\omega }{\tau }_{g}(\omega )$$

7 $$TOD(\omega )=\frac{{d}^{2}}{d{\omega }^{2}}{\tau }_{g}(\omega )$$

where ${\tau }_{g}$ is the group delay, equivalent to the value ${\rm{\Delta }}{t}_{out}$ at the given frequency component and *θ*_*d*_ is the frequency-dependent first-order diffraction angle.

Figure 2b–d show the comparison of GD, GDD and TOD results obtained from our modified matrix method, the original Kostenbauder matrix and analytical expression^29, 30^. Our analysis method shows good agreement with the result of the analytical solution, which exhibits high-order dispersion. In contrast, when using the Kostenbauder matrix, the accuracy of the results is limited to second-order dispersion where the analyzed GD is linearly related to the frequency. Due to the limitation, the Kostenbauder matrix method results in a constant GDD and a zero high-order dispersion (TOD). We note that evaluation of higher-order dispersion using our method is limited only by the floating point accuracy of numerical differentiation.

### Matrix Analysis of Time/Warped Stretch Imaging System

**Figure 3:**
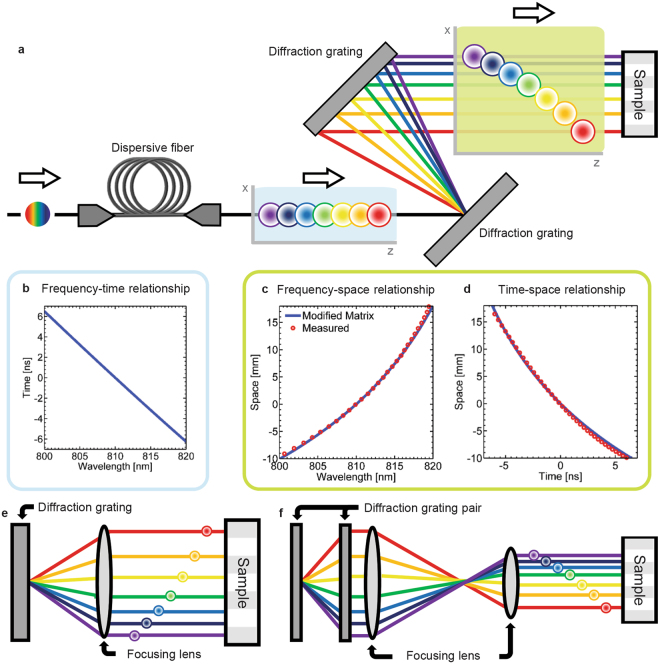
Matrix analysis of time stretch imaging system. **(a)** Schematic of time stretch imaging system (laser scanner-type). Ultrashort optical pulses with a center wavelength of 810 nm and an optical bandwidth of 20 nm is generated by a Ti:Sapphire mode-locked laser. The group delay dispersion of the dispersive fiber is −650 ps/nm and the groove density of the diffraction grating pair is 2200 lines/mm. The light blue and light green bounded graphs represent the spectro-spatio-temporal maps of the optical pulse after the dispersive fiber and after the diffraction grating, respectively. The temporal position is t = −z/c (z: propagation direction and c: velocity of light) (**b**) Frequency-to-time mapping function ${t}_{\omega }(\omega )$ after the dispersive fiber. (**c**) Frequency-to-space relationship *x*_*ω*_(*ω*) and (**d**) time-to-space mapping function *x*_*t*_(*t*) after the diffraction grating. Different types of spatial dispersion configuration are shown in (**e**) and (**f**). Either transmission or reflection type diffraction gratings can be used in any of these configurations.

We employ the matrix analysis method for a laser scanning type time stretch imaging system^11^ shown in Fig. 3a. The operation of imaging entails optical dispersion in time and space domain. A broadband optical pulse is temporally dispersed with dispersive fiber in the TS-DFT process. It is then spatially dispersed by a pair of diffraction grating into a one-dimensional rainbow. After being respectively mapped in the temporal and spatial domain, the pulse is incident on imaging target and performs the scan. The schematic of the imager in Fig. 3a is expressed mathematically as

8 $$[\begin{array}{c}{\rm{\Delta }}{x}_{out}\\ {\rm{\Delta }}{\theta }_{out}\\ {\rm{\Delta }}{t}_{out}\\ {\rm{\Delta }}{f}_{out}\end{array}]={M}_{total}\cdot [\begin{array}{c}{\rm{\Delta }}{x}_{in}\\ {\rm{\Delta }}{\theta }_{in}\\ {\rm{\Delta }}{t}_{in}\\ {\rm{\Delta }}{f}_{in}\end{array}]\quad ({M}_{total}={M}_{grating}\cdot {M}_{freespace}\cdot {M}_{grating}\cdot {M}_{fiber}).$$

Figure 3b shows the frequency-to-time mapping established by the propagation of the pulse through a dispersive fiber. Figure 3c and d show the frequency-to-space and time-to-space mappings after passing through the diffraction grating pair. The numerical results obtained by our matrix analysis agree well with the measured data from our previous study^11^. We note that different types of spatial dispersion configurations are possible as shown in Fig. 3e and f. The total optical matrix for each cases can be simply derived by multiplication of the corresponding optical matrices (i.e., focusing lens and free-space).

Several important parameters can be directly obtained from the overall frequency-space-time mapping resulting from the matrix evaluation; these include the overlap condition between consecutive scanning pulses, field-of-view (FOV), total number of pixels, detection sensitivity, and the explicit representation of the reconstruction map^2, 22^. When the scan rate of time stretch imaging system is represented by *R*, the temporal window for the time stretched pulse is limited by the pulse repetition period of *R* ^−1^. In order to avoid overlap between consecutive pulses the following condition needs to be satisfied.

9 $${\rm{\Delta }}\tau  < {R}^{-1}$$

10 $${\rm{\Delta }}\tau =|{t}_{\omega }({\omega }_{low})-{t}_{\omega }({\omega }_{high})|$$

where ${\rm{\Delta }}\tau$ is the time stretched pulse width, ${t}_{\omega }$ is the frequency-time relationship obtained from the matrix analysis as shown in Fig. 3b, ${\omega }_{low}$ and ${\omega }_{high}$ are the cutoff optical frequencies at lowest and highest end of the spectrum respectively. The FOV of the system is determined by the spectral width of the pulse and frequency-to-space mapping of the spatial dispersion component. The FOV is given by

11 $${\rm{\Delta }}{x}_{FOV}=|{x}_{\omega }({\omega }_{low})-{x}_{\omega }({\omega }_{high})|$$

where ${x}_{\omega }$ is the frequency-space relationship as shown in Fig. 3c. The number of pixel *N* is equivalent to the number of temporally sampled points in the digitizer, which is expressed as

12 $$N={f}_{dig}{\rm{\Delta }}\tau $$

where *f*_*dig*_ is the sampling rate of the digitizer. The detection sensitivity of the imager is largely determined by the fundamental noise mechanisms (shot noise, dark current noise and thermal noise) of the photodetector^22^ and the frequency-to-time mapping profile. Assuming far field conditions for frequency-to-time mapping^13, 21^, the number of collected photoelectrons at the pixel corresponding to the frequency *ω* is

13 $${P}_{in}(\omega )\cong \eta \cdot S(\omega )\cdot {(\frac{d{t}_{\omega }(\omega )}{d\omega })}^{-1}/{f}_{dig}$$

where *η* is the quantum efficiency of the photodetector, and *S*(*ω*) is the spectral density of incident photon flux. Without loss of generality, considering both dispersive Fourier transformation with Raman amplification and without, the total noise of the imaging system becomes^22, 31^,

14 $${\sigma }_{total}(\omega )=\sqrt{{(G(\omega )\cdot Q\cdot {\sigma }_{s}(\omega ))}^{2}+{{\sigma }_{d}}^{2}+{{\sigma }_{T}}^{2}}$$

where *G*(*ω*) is the optical amplification spectrum, *Q* is the amplification noise figure, ${\sigma }_{s}(\omega )$ is the shot noise which can be approximated by ${\sigma }_{s}(\omega )={P}_{in}{(\omega )}^{1/2}$, ${\sigma }_{d}$ is the dark current noise and ${\sigma }_{T}$ is the thermal noise. With the signal and noise defined, we may express signal-to-noise ratio (SNR) as:

15 $$SN{R}_{\omega }(\omega )=\frac{G(\omega )\cdot {P}_{in}(\omega )}{{\sigma }_{total}(\omega )}=\frac{{P}_{in}(\omega )}{\sqrt{{(Q\cdot \sqrt{{P}_{in}(\omega )})}^{2}+{(\frac{{\sigma }_{d}}{G(\omega )})}^{2}+{(\frac{{\sigma }_{T}}{G(\omega )})}^{2}}}$$

The spectral description of SNR (denoted by *SNR*_*ω*_(*ω*)) can be conveniently changed to its corresponding spatial description by $SN{R}_{x}(x)=SN{R}_{\omega }({\omega }_{x}(x))$. Finally, the reconstruction of image can be simply achieved by mapping the temporal position of the recorded signal to the spatial coordinate using the time-to-space mapping ${x}_{t}(t)$.

In the next section, we build a mathematical model of the time stretch imaging operation. In order to quantify the signal broadening (spatial resolution) of the imaging system, the optical and electrical response of the system needs to be mathematically considered. The optical mapping result from the matrix analysis (temporal dispersion profile *t*_*ω*_ and spatial dispersion profile *x*_*ω*_) is integrated to the mathematical model for simulating the optical wave propagation in the dispersive elements. At the final stage of the model, the time-to-space mapping *x*_*t*_ is used for reconstructing the image from the electrical scan signal.

### Mathematical Model

The imaging performance of the system is determined by the spectro-spatio-temporal map of the optical pulse in conjunction with the responses of diffraction grating pair, dispersive fiber, photodetector, and digitizer. In order to rigorously investigate the imaging process, we have established a mathematical model for the time stretch imaging system (laser scanner type)^11^.

We assume the initial optical pulse to be a transform-limited Gaussian pulse with its electrical field in the frequency domain expressed as

16 $${E}_{1}(\omega )={E}_{0}{(2\pi {T}_{0})}^{1/2}\exp (\frac{-{\omega }^{2}{{T}_{0}}^{2}}{2})$$

where *E*_0_ is the pulse amplitude and *T*_0_ is the pulse half-width. The pulse after propagation through the dispersive fiber with frequency response ${H}_{fiber}(\omega )$ is expressed as

17 $${E}_{2}(\omega )={H}_{fiber}(\omega )\cdot {E}_{1}(\omega )$$

18 $${H}_{fiber}(\omega )=\exp \,[i\int {\tau }_{g}(\omega )d\omega ]=\exp \,[i\int {t}_{\omega }(\omega )d\omega ]$$

where ${\tau }_{g}(\omega )$ is the group delay of the dispersive element. Due to TS-DFT, the group delay is identical to the frequency-time relationship ${t}_{\omega }(\omega )$ obtained from the matrix analysis. We note that the group-delay dispersion of the dispersive fiber is required to meet the far-field condition^13, 21^ to successfully map the spectrum into time domain. The diffraction grating pair spectrally encodes the spatial information of the sample ${S}_{x}$ where the process can be viewed as amplitude spectral mask with spectrally varying broadening profile $g(\omega ,\omega ^{\prime} )$ for each frequency component *ω*′. The frequency response of the diffraction grating pair and imaging target can be calculated as

19 $${H}_{grating-sample}(\omega )=\int {S}_{\omega }(\omega ^{\prime} )\cdot g(\omega ,\omega ^{\prime} )d\omega ^{\prime} $$

where ${S}_{\omega }(\omega )={S}_{x}({x}_{\omega }(\omega ))$, which is the spectral representation of ${S}_{x}$ obtained by a change of domain using the frequency-to-space mapping function ${x}_{\omega }(\omega )$. We have assumed that each spectral component to be a Gaussian beamlet when incident on the sample. The finite beam width of the Gaussian spectral component *ω*′ results in a Gaussian spectral broadening profile $g(\omega ,\omega ^{\prime} )$, which can be expressed as

20 $$g(\omega ,\omega ^{\prime} )=\exp [-2\,\mathrm{ln}\,2{(\frac{\omega -\omega ^{\prime} }{{w}_{\omega }(\omega \text{'})})}^{2}]$$

where spectral width ${w}_{\omega }(\omega )={w}_{x}\cdot {[d{x}_{\omega }(\omega )/d\omega ]}^{-1}$ is a product of the full width half maximum (FWHM) incident beam width of the spectral component ${w}_{x}$ on the sample and the inverse derivative of frequency-space relationship ${x}_{\omega }(\omega )$. In the case of the 2-f configuration shown in Fig. 3e (grating – focusing lens – sample), the beam width on the sample plane becomes ${w}_{x}=\sqrt{2\,{\rm{l}}{\rm{n}}(2)}F{\lambda }_{0}\,\cos \,{\theta }_{i}/(\pi W\,\cos \,{\theta }_{d})$ where *W* is the input beam waist, *F* is the focal length of the lens after the diffraction grating, *λ*_0_ is the optical wavelength, *θ*_*i*_, and *θ*_*d*_ are the incident and diffraction angle respectively. Here, the beam width ${w}_{x}$ can be numerically determined by performing Finite Difference Time Domain simulations when using unconventional spatial dispersion devices. Using the derived frequency response, the electric field after the diffraction grating pair and the sample becomes

21 $${E}_{3}(\omega )={H}_{grating-sample}(\omega )\cdot {E}_{2}(\omega )$$

The transmitted or reflected stream of light from the sample is then converted into the electrical signal by the photodetector and recorded by the digitizer. The photocurrent ${I}_{P}(t)$ and the frequency domain representation of the photocurrent ${I}_{e}(\omega )$ after photodetection and digitization becomes

22 $${I}_{P}(t)=K{E}_{3}(t){E}_{3}^{\ast }(t)$$

23 $${I}_{e}(\omega )={H}_{e}(\omega )\cdot {I}_{P}(\omega )$$

where *ω* now represents electrical signal (RF) frequency, *K* is the responsivity of the photodetector and ${H}_{e}(\omega )$ is the frequency response of the electrical back-end consisting of photodetector and digitizer. We may either use an equivalent RC circuit model^2, 21^ or the frequency response acquired from the actual photodetector for ${H}_{e}(\omega )$. Finally, the signal is digitally sampled and recorded by the digitizer as

24 $${I}_{dig}[n]\propto {I}_{e}(t)\cdot \delta (t-nT)$$

where *T* is the sampling period of the digitizer and *n* is the pixel index of the recorded scan signal. Each pixel of the recorded image signal ${I}_{dig}[n]$ is located at its corresponding discrete spatial coordinate $X[n]$, which can be expressed as

25 $$X[n]={x}_{t}(t)\cdot \delta (t-nT)$$

Finally, the imaging result ${I}_{x}[X]$ is reconstructed by mapping the signal ${I}_{dig}[n]$ to the spatial domain $X[n]$.

## Results and Discussion

In this section, we consider the time stretch imaging system used in ref. 11 to demonstrate our analysis method. First, we perform numerical simulations and compare the results with the experimental data to validate our method. We then analyze the spatial resolution in terms of line-spread function (LSF) and modulation transfer function (MTF), which are widely used metrics for evaluating imaging systems. By taking note of the scan signal warp in the imaging system^11^, we also discuss the effects of high-order spatial dispersion on the resultant image.

### Simulation of Time/Warped Stretch Imaging

**Figure 4:**
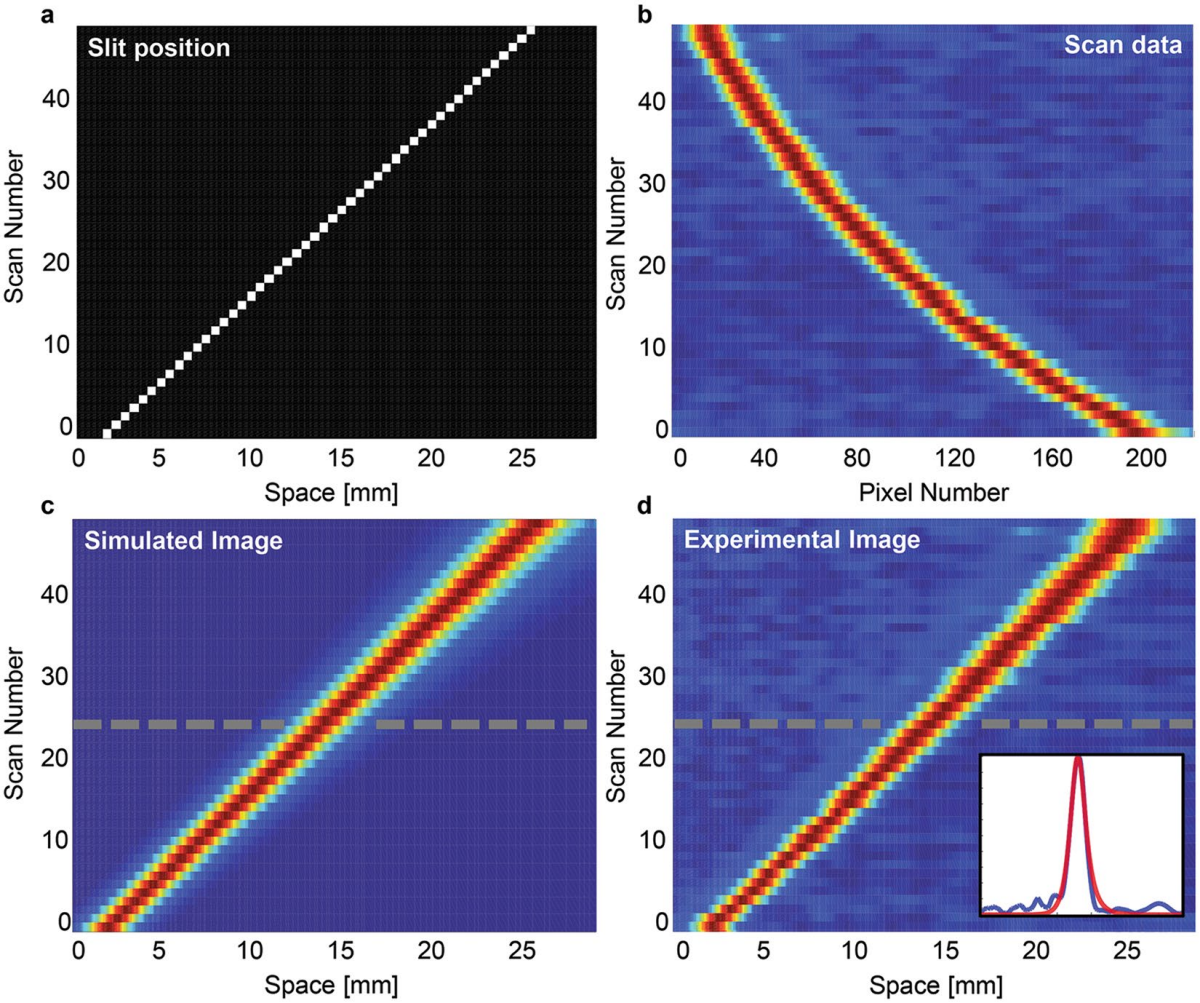
Reconstruction and numerical simulation of time stretch imaging system from ref. 11. **(a)** A 0.5 mm wide slit is placed immediately before the target sample and translated transversely along the scanning beam at intervals of 0.5 mm. The total number of scans is 49. The FOV of the system is limited to 24.5 mm, since the time stretched pulse width is limited by 11 ns to avoid overlapping of the consecutive pulses. **(b)** Recorded scan data from the digitizer^11^. **(c)** Reconstructed image from simulation. **(d)** Reconstructed image from the experimental scan data. The inset shows a comparison of the reconstructed image from the simulation (solid red) and from experimental data (solid blue). The 24th scan number is indicated by the dashed grey line.

Discrete-time complex envelope analysis^21^ was used for the numerical simulation of the mathematical model. We have compared and analyzed the imaging result of simulation and experiment from ref. 11 and the results are plotted in Fig. 4. As shown in Fig. 4a, total of 49 scans were performed while 0.5 mm wide slit was translated by interval of 0.5 mm for each scan. The transmitted light through the slit is captured and recorded by the photodetector and digitizer. The recorded scan from the previous experiment^11^ is shown in Fig. 4b which displays a nonlinear relationship between time (pixel number) and space. We reconstruct the image by mapping the signal to corresponding spatial coordinate (eq. 25) where the result is shown in Fig. 4d. We can see that the reconstructed image from the simulation Fig. 4c matches well with the image from the experiment. In addition, we have observed the broadening feature of the image across the FOV as shown in Fig. 4c and d which implies nonuniform spatial resolution of the image.

### Spatial Resolution

The spatial resolution of the imager is defined by a combination of factors, including the diffraction grating, dispersive fiber, photodetector and digitizer. We summarized the spatial resolution limits for each contributing factors in Table 2. The spatial resolution limit of the diffraction grating can be directly expressed with the beam width *w*_*x*_ of a spatially dispersed frequency component at the imaging target, whereas the spectral ambiguity of the time stretch process (TS-DFT)^13^ for the dispersive fiber is translated into a spatial resolution by the frequency-to-space conversion factor $|d{x}_{\omega }(\omega )/d\omega |$. We note that the far-field condition^13, 21^ should be satisfied for the spatial resolution expression to be valid for time stretch processes. The temporal resolution which arises from the finite electrical bandwidth of the detection system also limits the spatial resolution. We define the temporal resolution ${\tau }_{FWHM}$ as the FWHM of detection system’s temporal impulse function and convert it into spatial resolution by using frequency-to-time $|d{t}_{\omega }(\omega )/d\omega |$ and frequency-to-space $|d{x}_{\omega }(\omega )/d\omega |$ conversion factors, respectively. In a similar manner, the resolution limit arises from the temporal sampling rate of the digitizer ${f}_{dig}$ ^2^. Here, the spatial resolution limits for each factor are derived as a function of optical frequency.

**Table 2:** Spatial resolution limit.

Element	Spatial resolution
Diffraction grating	*w*_*x*_
Dispersive fiber	$|\frac{d{x}_{\omega }(\omega )}{d\omega }|\cdot \sqrt{\frac{4\pi }{|\tfrac{d{t}_{\omega }(\omega )}{d\omega }|}}$
Electrical bandwidth	$|\frac{d{x}_{\omega }(\omega )}{d\omega }|\cdot \frac{{\tau }_{FWHM}}{|\tfrac{d{t}_{\omega }(\omega )}{d\omega }|}$
Sampling rate	$|\frac{d{x}_{\omega }(\omega )}{d\omega }|\cdot \frac{1}{{f}_{dig}\cdot |\tfrac{d{t}_{\omega }(\omega )}{d\omega }|}$

The overall spatial resolution can be obtained by evaluating the net response of the system. In the spatial domain, LSF represents the net broadening caused by the imaging system^32, 33^. To obtain the LSF, we perform imaging simulation of Dirac delta function as the imaging sample. As previously shown, since the spatial resolution is not uniform over the scanning beam, the LSF should also be considered as a shift-variant and locally defined function. Therefore, we take series of $LSF(x,x^{\prime} )$ corresponding to imaging simulation result of Dirac delta function $\delta (x-x\text{'})$ where $x^{\prime}$ is the spatial coordinate of interest within the FOV.

**Figure 5:**
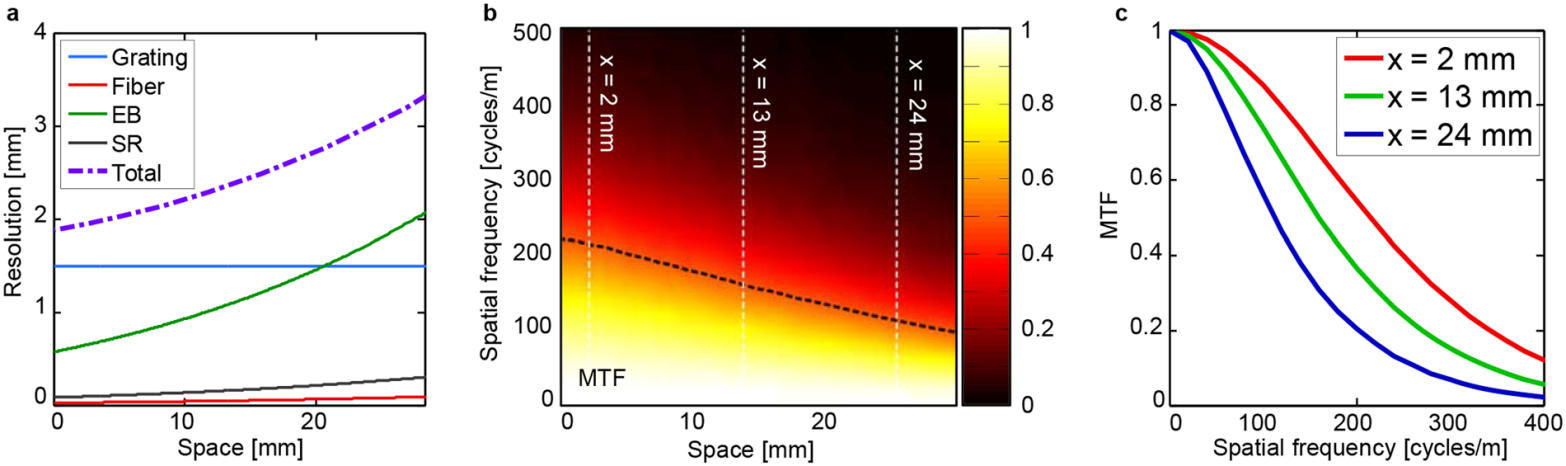
LTF and MTF spatial resolution analysis of time stretch imaging system in ref. 11. The temporal response of the detection system was assumed to have a rise-time of 750 ps, and a sampling rate of 20 GS. (**a**) The spatial resolution of a single line scan. Grating: diffraction grating pair; Fiber: dispersive fiber; EB: electrical bandwidth; SR: sampling rate; Total: overall spatial resolution as defined by the FWHM of the LSF. (**b**) Three-dimensional color plot of MTF. The black-dashed line represents the MTF cut-off frequency at half-maximum. **(c)** MTF as evaluated for x = 2, 13, 24 mm each corresponding to the white-dashed lines in (**b**).

In this study, we define the overall spatial resolution as the FWHM of the LSF. As shown in Fig. 5a as a purple-dotted line, the total spatial resolution clearly exhibits a nonuniform profile along the line scan. When assessing the contributions from each individual optical elements, we find that the spatial resolution is primarily limited by the diffraction grating and the photodetector. While the resolution profile of the diffraction grating pair is constant and equal to the incident beam width (1.5 mm). The nonlinear frequency-to-space mapping introduced by the diffraction grating pair causes the resolution profile of the other optical elements to vary across the FOV.

Another widely used metric for spatial resolution of an imaging system is the MTF, which represents the resolving power in terms of spatial frequency^32, 33^. Like the LSF, the MTF is also shift-variant and should be also determined independently at each spatial position $x^{\prime}$. The locally - defined MTF is evaluated by taking the Fourier transform of the LSF at the corresponding location. It can be expressed as

26 $$MTF({f}_{x},x\text{'})=k|{F}\,[LSF(x,x\text{'})]|$$

where ${f}_{x}$ is the spatial frequency, *k* is the normalization constant and ${F}$ is the Fourier transform operator. Figure 5b shows the three-dimensional color plot of the MTF defined at each spatial position $x^{\prime}$. As we move away from the spatial position x = 0, the FWHM of the LSF (purple-dotted line in Fig. 5a) increases while the MTF bandwidth (black-dashed line in Fig. 5b) decreases. This means that a smaller line broadening (LSF) allows for the detection of features at a higher spatial frequency (i.e., MTF bandwidth). The correlation is also evident from the Fourier transform relationship between the MTF and the LSF in eq. (26). Figure 5c shows the calculated MTFs for three different locations as denoted by white dashed lines in Fig. 5b. When imaging a sinusoidal pattern sample with a single spatial frequency, the contrast of the imaged pattern will be different across the FOV. The sinusoidal image obtained at spatial positions x = 2, 13, 24 mm will have different contrast proportional to their MTF (Fig. 5c) shown as red, green and blue solid lines, respectively.

### Influence of Spatial Dispersion Element

**Figure 6:**
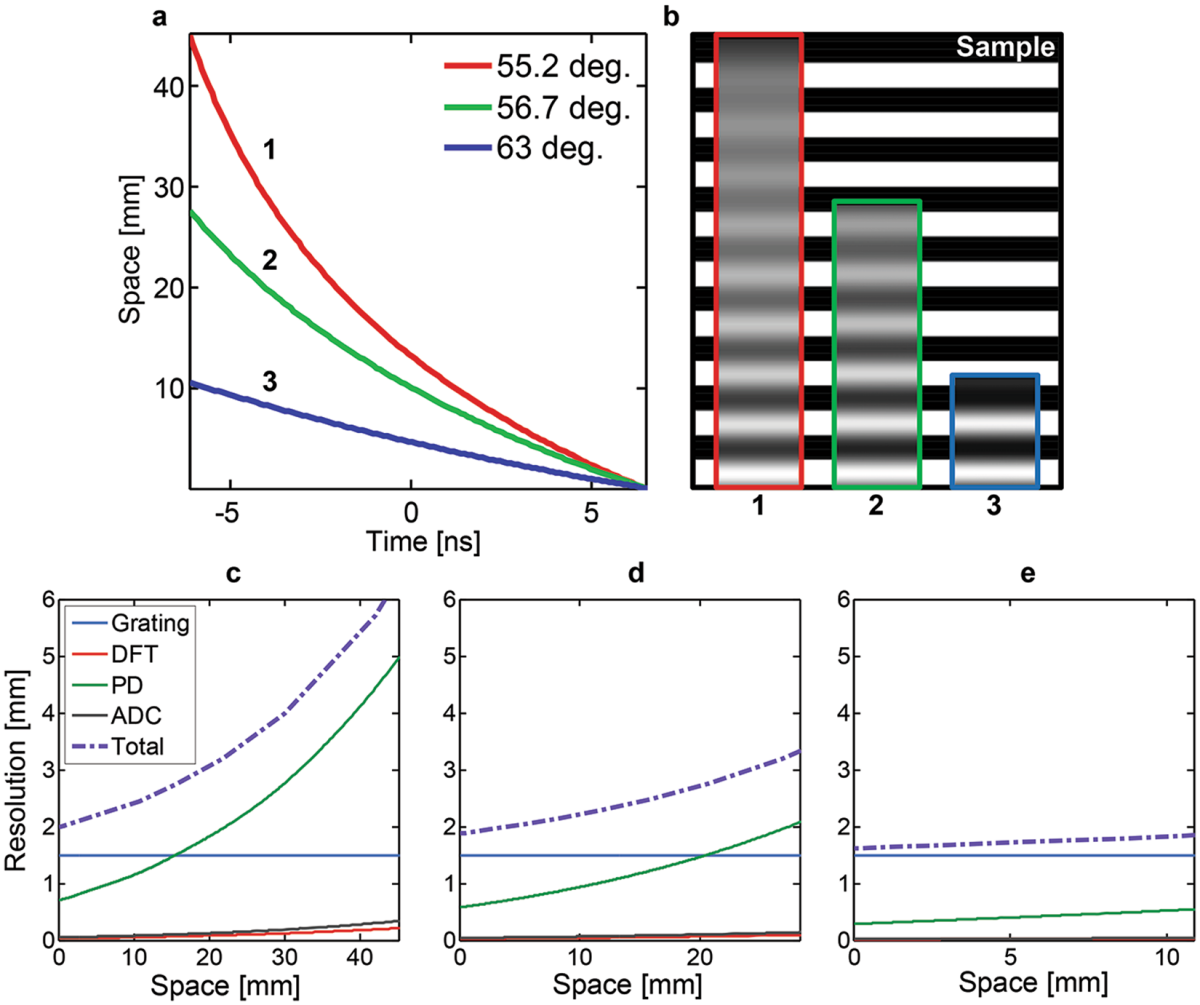
The effects of incidence angle on the space-to-time mapping and spatial resolution. (a) The space-to-time mapping at three different incident angles 55.2° (“1”, solid red), 56.7° (“2”, solid blue), 63° (“3”, solid green). (**b**) The reconstructed images from numerical simulation at the three incidence angles. The 5-mm period picket fence pattern used as the sample is shown in the background for reference. (**c,d**, and **e**) Spatial resolution profiles at the three incidence angles. The FOV is 45.1 mm, 28.2 mm, and 10.9 mm at each respective angle, respectively.

Time/warped stretch imaging systems generally have fixed temporal dispersion elements, each of which is governed by their respective dispersion profiles and the propagation length within each dispersive element. However, the spatial dispersion of diffraction grating pairs can be adjustable simply by repositioning the gratings. For example the FOV is proportional to the distance between the grating pair. In particular, changing the incident angle of the optical input into the diffraction grating affects the FOV and spatial resolution profile (warp profile) simultaneously. To illustrate this effect in a quantitative fashion, we numerically analyze the FOV and spatial resolution of the imaging system for three different incident angles (55.2°, 56.7° and 63°) at the diffraction grating pair used in ref. 11. The angular tuning of the diffraction grating results in varying frequency-space relationship *x*_*ω*_ and time-space relationship *x*_*t*_ as shown in Fig. 6a. It shows that a mere difference of 8 degrees in the incident angle yields a more than four-fold difference in the FOV. Similarly, the degree of nonlinearity in the time-to-space mapping function is also affected by changes in the incident angle, being the most significant for the incident angle of 55.2° and the least significant for the Littrow configuration (63°). This feature is evident from the grating equation where spatial (angular) dispersion becomes more nonlinear as we move away from the Littrow angle. Figure 6b shows a comparison of the reconstructed images at the three specified angles. We used a picket fence pattern with a period of 5 mm as the imaging sample. Visual inspection of the edge sharpness indicates that the nonlinearity in the space-time relationship promotes an increasing spatial resolution along the scan line. A detailed analysis of the spatial resolution is shown in Fig. 6c–e for each incidence angle, respectively. As mentioned in the spatial resolution analysis, the spatial resolution imposed by the diffraction grating pair is fixed by the incident beam width, which is identical for all cases. However, the nonlinear spatial dispersion enhances the varying amount of $|d{x}_{\omega }(\omega )/d\omega |$ over the spectrum which boosts the nonuniformity in spatial resolution limits of other elements (cf. Table 2). In addition, the spatial resolution becomes coarser with wider FOV which results from high average value of $|d{x}_{\omega }(\omega )/d\omega |$. The analysis results display that the reconfiguration of spatial dispersion component can significantly influence the FOV and spatial resolution profile of the acquired image. The above analysis shows that the physical adjustments to the spatially dispersive elements, such as in the case of angular tuning of the incident angle of the optical beam upon the grating pair, may provide an additional pathway for designing the spectrotemporal mapping profile of warped stretch transform imaging systems.

## Conclusions

Here, we have presented a general analysis method for characterizing arbitrary spatial, spectral and temporal dispersion profiles, the crucial components used in warped stretch imaging systems. We have established the modified matrix method to accommodate arbitrary frequency-space-time map pings of dispersive optical systems and constructed a mathematical model to numerically simulate the imaging operation. Employing our numerical analysis, we have quantified the spatial resolution in terms of its LSF and MTF, and the design parameters such as the FOV, total number of pixels, detection sensitivity and reconstruction map. Finally, we investigated the effect of tuning the nonlinear spatial dispersion on the imaging operation. Our method serves as a comprehensive tool for characterizing arbitrary reconstruction mappings and for empirically optimizing the performance of actual warped stretch imaging systems, and enables the ability to fully design a warped stretch imaging system prior to its construction. We anticipate that the increased accessibility to warped stretch systems that this method provides would encourage its adoption for precision imaging and ultrafast optical inspection applications.

### Data availabilty

The datasets generated and analyzed during the current study are available from the corresponding author on reasonable request.
